# RRx-001 Exerts Neuroprotection Against LPS-Induced Microglia Activation and Neuroinflammation Through Disturbing the TLR4 Pathway

**DOI:** 10.3389/fphar.2022.889383

**Published:** 2022-04-06

**Authors:** Jie Fang, Jing She, Fang Lin, Jun-Chao Wu, Rong Han, Rui Sheng, Guanghui Wang, Zheng-Hong Qin

**Affiliations:** ^1^ Department of Pharmacology and Laboratory of Aging and Nervous Diseases, Jiangsu Key Laboratory of Neuropsychiatric Diseases, College of Pharmaceutical Sciences, Soochow University, Suzhou, China; ^2^ Department of Pharmacology and Laboratory of Molecular Pathology, Jiangsu Key Laboratory of Neuropsychiatric Diseases, College of Pharmaceutical Sciences, Soochow University, Suzhou, China

**Keywords:** RRx-001, neuroinflammation, TAK1, NF-κB, NLRP3 inflammasome, MAPK, oxidative stress

## Abstract

Neuroinflammation plays an important role in the pathogenesis of many central nervous system diseases. Here, we investigated the effect of an anti-cancer compound RRx-001 on neuroinflammation and its possible new applications. BV2 cells and primary microglia cells were used to evaluate the role of RRx-001 in LPS-induced microglial activation and inflammatory response *in vitro*. And, we found that the increase in the synthesis and release of cytokines and the up-regulation of pro-inflammatory factors in LPS-treated microglial cells were significantly reduced by RRx-001 pretreatment. As the most classical inflammatory pathways, NF-κB and MAPK signaling pathways were activated by LPS, but were inhibited by RRx-001. Transcription of NLRP3 was also reduced by RRx-001. In addition, LPS induced oxidative stress by increasing the expression of Nox mediated by transcription factors NF-κB and AP-1, while RRx-001 pretreatment ameliorated Nox-mediated oxidative stress. LPS-induced activation of TAK1, an upstream regulator of NF-κB and MAPK pathways, was significantly inhibited by RRx-001 pretreatment, whereas recruitment of MyD88 to TLR4 was not affected by RRx-001. LPS-primed BV2 condition medium induced injury of primary neurons, and this effect was inhibited by RRx-001. Furthermore, we established a neuroinflammatory mouse model by stereotactic injection of LPS into the substantia nigra pars compacta (SNpc), and RRx-001 dose-dependently reduced LPS-induced microglial activation and loss of TH + neurons in the midbrain. In conclusion, the current study found that RRx-001 suppressed microglia activation and neuroinflammation through targeting TAK1, and may be a candidate for the treatment of neuroinflammation-related brain diseases.

## Introduction

In the central nervous system, neuroinflammation characterized by activation of microglia cells is one of the important causes of almost all neurodegenerative diseases and brain cancer (Glass et al., 2010; Yan et al., 2014; Mostofa et al., 2017; Candido et al., 2021; Vandenbark et al., 2021). As the most important immune cells in the brain, microglia, which are homologous to peripheral macrophages, have been recognized to be involved in neuroinflammatory responses in brain pathophysiology (Hickman et al., 2018; Stephenson et al., 2018). Under physiological conditions, microglia, which are evenly distributed in the brain parenchyma as nurturer and sentinel, play a housekeeping role by constant moving and sensing the microenvironment in the central nervous system, including synaptic remodeling, maintaining milieu homeostasis, migrating into the sites of neuronal death to remove dead or dying cells, and releasing nutritional factors to maintain the normal function of neurons (Hanisch and Kettenmann, 2007). Once microglia are activated, neuroinflammation, characterized by the abnormal release of inflammatory cytokines, is induced to damage neurons and exacerbate the course of diseases (Xanthos and Sandkühler, 2013). Therefore, new drugs targeting the activation of microglia and inhibiting the inflammatory response have become an urgent subject to study.

During the inflammatory response, LPS (lipopolysaccharide), most commonly used for activation of microglia, triggers multiple inflammatory signaling pathways by binding to toll-like receptor 4 (TLR4), to activate NF-κB and MAPK pathways (Dauphinee and Karsan, 2005; Lu et al., 2008; Gay et al., 2014). In the cascade of TLR4 activation, the TGF β-activated kinase 1 (TAK1) is an upstream kinase involved in the activation of both NF-κB and MAPK pathways. Upon activation of TAK1, the kinase IKK is phosphorylated, and the activated IKK induces the phosphorylation and degradation of IкB-α, then free NF-κB translocates into the nucleus to initiate the transcription of pro-inflammatory factors and NLRP3 inflammasome (Hayden and Ghosh, 2008; Bauernfeind et al., 2009). Moreover, as a mitogen-activated protein kinase kinase (MAP3K), TAK1 induces the transcription factor AP-1 translocation into the nucleus by activating MAP kinases, such as ERK1/2, JNK1/2, and p38 MAPK, which are involved in the transcription of various pro-inflammatory factors (Herdegen and Waetzig, 2001; Schonthaler et al., 2011). Meanwhile, The occurrence and development of neuroinflammation are also promoted by ROS derived from the NADPH oxidase family, especially Nox1, Nox2, and Nox4 (Kang et al., 2001; Cheret et al., 2008; Barron et al., 2017; Thummayot et al., 2018; Fan et al., 2020). It is reported that activated NF-κB and MAPK signaling pathways promote transcription of Nox isoforms (Anrather et al., 2006; Huo et al., 2011). Therefore, TAK1-induced NF-κB/NLRP3 inflammasome and MAPK pathways and Nox-derived oxidative stress may be very effective targets for the treatment of neuroinflammation.

RRx-001, derived from the explosive propellant 1,3,3-trinitroazetidine (TNAZ) structure of guns and rockets, is a non-explosive derivative obtained by removing a nitro group (NO_2_) and replacing the bromoacetate group with TNAZ (Sikder et al., 2004). Subsequent studies have shown that RRx-001 exerts radiation sensitivity in hypoxic cells, and has been shown a good synergistic effect on tumor tissue when combined with radiation therapy in hypoxic tumor cells (Ning et al., 2012). In phase I and II clinical trials, RRx-001 combined with chemotherapy drugs demonstrated good therapeutic effects on several types of tumors, including multiple myeloma (Das et al., 2016), small-cell lung cancer (Morgensztern et al., 2019), and colon cancer (Zhao et al., 2017). In addition, RRx-001 can reduce leukocyte adhesion against ischemic reperfusion injury (Cabrales et al., 2017), and can also inhibit inflammatory response when combined with artemether in the treatment of conditioned medium (CM) (Yalcin et al., 2015). Recently, RRx-001 has been identified as a potent covalent NLRP3 inhibitor to ameliorate inflammatory diseases (Chen et al., 2021). Rongbin Zhou et al. emphasized that RRx-001 inhibited the assembly of NLRP3 inflammasomes at low doses (300 nM) by covalently binding to the 409 cysteine of NLRP3 and blocking the interaction between NLRP3 and NEK7 (Chen et al., 2021). However, whether RRx-001 can inhibit neuroinflammation induced by LPS and involves in other mechanisms, particularly at higher doses? Whether inhibition of neuroinflammation by RRx-001 contributes to neuroprotection of RRx-001?

In this study, we aimed to investigate if RRx-001 inhibits LPS-induced neuroinflammation and protects dopaminergic neurons and its molecular target. Our results demonstrated for the first time, that in LPS-induced neuroinflammatory models, RRx-001 exhibited neuroprotective effects *in vitro* and *in vivo* by inhibiting microglia activation and neuroinflammation, and the mechanism may be related to the disturbance of TLR4 mediated pathway and oxidative stress.

## Materials and Methods

### Reagents

RRx-001 was purchased from MedChemExpress (Monmouth Junction, NJ, United States) and dissolved in dimethyl sulfoxide (DMSO) for cell experiments, and dissolved in 10% DMSO and 90% corn oil for animal experiments. Lipopolysaccharide (LPS) and ATP were purchased from Sigma (St. Louis, United States) and dissolved in PBS for cell experiments, and dissolved in normal saline for animal experiments. DCFH-DA (2′, 7′-dichlorodihydrofluorescein diacetate) was purchased from Solarbio (Beijing, China). Propidium iodide (PI), Hoechst 33,342, and DAPI (4′, 6-diamidino-2-phenylindole) were purchased from Beyotime Biotechnology (Shanghai, China).

### Animal Experiments

Male C57BL/6 mice, 6–8 weeks, 22–28 g, were purchased from the Experimental Animal Center of Soochow University. All experimental animals in this study were approved by the Animal Ethics Committee of Soochow University and followed the regulations on the management of experimental animals issued by the Animal Committee of Soochow University. According to the experimental arrangement, the mice were randomly divided into six groups (n = 6): control (normal saline), RRx-001 (10 mg/kg), LPS (1 mg/kg), LPS + RRx-001 (2.5 mg/kg), LPS + RRx-001 (5 mg/kg), LPS + RRx-001 (10 mg/kg). All RRx-001 treatment groups were intraperitoneally (i.p.) injected with corresponding dose once a day for seven consecutive days, while the other groups were intraperitoneally injected with the equal volume of vehicle. On the day 8, all LPS treatment groups were given with LPS (1 mg/kg) by stereotactic injection in the bilateral substantia nigra pars compacta at AP - 3.3 mm, DV - 4.6 mm, ML ± 1.2 mm from Bregma.

### Cell Culture

BV2 cells, a microglia cell line derived from mouse, were cultured in Dulbecco’s Modified Eagle Medium (DMEM) from Gibco BRL (Grand Island, NY, United States) with 10% fetal bovine serum and 1% penicillin/streptomycin. Primary glial cells were obtained in a clean environment and cultured in Dulbecco’s Modified Eagle Medium Nutrient Mixture F-12 (DMEM/F-12) with 10% 55°C-inactivated fetal bovine serum and 1% penicillin/streptomycin in 75 cm^2^ cell culture flask (Corning Incorporated, Corning, United States). After 7 days of culture, primary microglia cells were obtained from the supernatant of culture flask by shaking at 37°C and 240 rpm for 2 h, and then were cultured in a cell culture plate for experiments. The adherent primary astrocytes were detached with trypsin and then collected by centrifugation, and planted in the cell culture plate with appropriate density for experiments. Primary neurons were isolated from the cerebral cortex of embryos dissected from pregnant mice at gestation day 17 in a clean environment and cultured in Neurobasal™ Plus medium from Gibco BRL (Grand Island, NY, United States) with glutamine, B-27 and 1% penicillin/streptomycin with appropriate density for experiments. All cells were cultured in an incubator with a temperature of 37°C, the humidity of 70–80%, and CO_2_ of 5%.

### Cell Viability Assay

Cell survival was measured by cell counting kit-8 (CCK-8) purchased from Dojindo (Kumamoto, Japan). 1×10^4^ BV2 cells, 2.5×10^4^ primary microglial cells, 2.5×10^4^ primary astrocytes and 5×10^4^ primary neurons were seeded in 96-well plates, respectively, and treated with RRx-001 at different concentrations with or without LPS (100 ng/ml) after cell adherence. After 24 h, the cells were incubated with 100 µl CCK-8 working solution for 2 h at 37°C. The absorbance was evaluated at 450 nm by using the infinite M1000 PRO multiscan spectrum (Tecan, Switzerland).

### Immunoblotting and Antibodies

The experimentally treated cells were collected and lysed in cell lysis buffer (1% sodium deoxycholate, 1% SDS, 1% Triton X-100, 150 mM NaCl and 10 mM Tris-HCl, pH 7.4) with a protease inhibitor cocktail (Roche, Switzerland). A phosphatase inhibitor cocktail is added to the cell lysis buffer to detect phosphorylated proteins. The intracellular proteins were isolated by SDS-PAGE and transferred to an NC (nitrocellulose) membrane. The NC membranes were blocked with 10% skimmed milk for 1 h and the proteins were analyzed by western blotting with antibodies. Antibodies against iNOS, COX-2, p65, IκB-α, JNK1/2, IKK-α, IL-1β, p-TAK1, NLRP3, caspase-1, and β-actin were purchased from Abcam (Cambridge, United States). Antibodies against p-p65, p-IκB-α, p-IKK-α, p-p38, p-ERK1/2, p-JNK1/2, p-Fos, ASC, TLR4, MyD88 and TAK1 were purchased from Cell Signaling Technology (Danvers, United States). Antibodies against p38, ERK1/2, Fos, Nox1, Nox2, Nox4, Histone 3, and G6PD were purchased from Santa Cruz Biotechnology (Santa Cruz, United States). The catalog numbers and concentrations used for all antibodies are shown in Table 1. The secondary antibodies, goat anti-rabbit or anti-mouse IgG (H + L), Alexa Fluor Plus 800, were from Thermo Fisher Scientific (Waltham, United States). The signals of protein bands were visualized with Odyssey Two-Color Infrared Fluorescence Imaging System (LI-COR Biosciences, United States).

**TABLE 1:** The catalog numbers and concentrations of the antibodies.

Antibody	Catalogue Number	Concentration
β-actin	ab8227	1: 5,000
COX-2	ab15191	1: 1,000
iNOS	ab15323	1: 1,000
IL-1β	ab216995	1: 1,000
IκB-α	ab32518	1: 1,000
p-p65	3033S	1: 1,000
p65	sc-71675	1: 1,000
IKK-α	61294S	1: 1,000
p-IKK-α	2697S	1: 500
JNK1/2	3,708	1: 1,000
p-IκB-α	2859	1: 1,000
p-TAK1	ab109404	1: 1,000
NLRP3	ab263899	1: 500
caspase-1	ab179515	1: 500
p-p38	4,511	1: 1,000
p-ERK1/2	4,370	1: 1,000
p-JNK1/2	9,255	1: 1,000
p-Fos	5,348	1: 1,000
ASC	13,833	1: 1,000
TLR4	14,358	1: 1,000
MyD88	4,283	1: 1,000
TAK1	5,206	1: 1,000
p38	sc-81621	1: 1,000
ERK1/2	4,695	1: 1,000
Fos	sc-398595	1: 1,000
Nox1	ab131088	1: 1,000
Nox2	sc-130543	1: 1,000
Nox4	ab133303	1: 1,000
Histone 3	4,299	1: 1,000
G6PD	sc-373887	1: 1,000
Iba1	019–19,741	1: 500
GFAP	MAB360	1: 500
TH	MAB152	1: 500

### Real-Time Quantitative PCR and Primers

After the total RNA was extracted from BV2 cells, primary microglial cells or tissues using RNAiso Plus reagent (Takara, Tokyo, Japan), the total RNA (500 ng) was reverse transcribed into cDNA using HiScript III first Strand cDNA Synthesis Kit (Vazyme, Nanjing, China). Real-Time Quantitative PCR was carried out using Taq Pro universal SYBR qPCR Master Mix (Vazyme, Nanjing, China) and ABI-7500 quantitative PCR system (Applied Biosystems, Warrington, United Kingdom). The sequence of PCR primers was shown in Table 2 (Gu et al., 2018).

**TABLE 2:** A list of primers sequences for RT-PCR analysis.

Gene	Forward	Reverse
m-β-actin	GACCTGACTGACTACCTC	GACAGCGAGGCCAGGATG
m-iNOS	TCC​CAG​CCT​GCC​CCT​TCA​AT	CGG​ATC​TCT​CTC​CTC​CTG​GG
m-COX-2	CAG​GCT​GAA​CTT​CGA​AAC​A	GCT​CAC​GAG​GCC​ACT​GAT​ACC​TA
m-TNF-α	CAT​CTT​CTC​AAA​ATT​CGA​GTG​ACA​A	TGG​GAG​TAG​ACA​AGG​TAC​AAC​CC
m-IL-6	GCT​ATG​AAG​TTC​CTC​TCT​GC	CTA​GGT​TTG​CCG​AGT​AGA​TC
m-Arg1	TTA​GGC​CAA​GGT​GCT​TGC​TGC​C	TAC​CAT​GGC​CCT​GAG​GAG​GTT​C
m-IL-10	GGC​AGA​GAA​CCA​TGG​CCC​AGA​A	AAT​CGA​TGA​CAG​CGC​CTC​AGC​C
m-CD206	TCA​GCT​ATT​GGA​CGC​GAG​GCA	TCC​GGG​TTG​CAA​GTT​GCC​GT
m-Nox1	GGT​TGG​GGC​TGA​ACA​TTT​TTC	TCG​ACA​CAC​AGG​AAT​CAG​GAT
m-Nox2	AGT​GCG​TGT​TGC​TCG​ACA​A	GCG​GTG​TGC​AGT​GCT​ATC​AT
m-Nox4	TTT​CTC​AGG​TGT​GCA​TGT​AGC	GCG​TAG​GTA​GAA​GCT​GTA​ACC​A
m-G6PD	CGA​GGC​CGT​CAC​CAA​GAA​C	GTA​GTG​GTC​GAT​GCG​GTA​GA

### Enzyme-Linked Immunosorbent Assay

1×10^4^ BV2 cells were seeded in 24-well plates, and pretreated with RRx-001 at proportionate doses of 0.2, 1 and 5 μM and 5×10^4^ primary microglial cells at doses of 0.2, 0.4 and 0.8 µM for 12 h, followed by LPS (100 ng/ml) for 24 h. After the cultured media were collected, the levels of TNF-α and IL-6 were measured by using a mouse TNF-α ELISA kit (Dakewe, Beijing, China) and mouse IL-6 ELISA kit (Boster Biological Technology co. ltd., Wuhan, China). Adhered cells were collected with trypsin and ultrasonicated to detect Nox4 activity with mouse NOX4 ELISA kit (Reddotbiotech, Kelowna, Canada). The absorbance was evaluated at 450 nm by using the infinite M1000 PRO multiscan spectrum (Tecan, Switzerland).

### Intracellular NADPH Quantification Assay

The concentration of NADPH was measured by EnzyChrom™ NADP^+^/NADPH assay kit purchased from BioAssay Systems (Hayward, United States). 1×10^4^ BV2 cells were seeded in 24-well plates, and treated with LPS (100 ng/ml) with or without RRx-001 (5 μM). After 24 h, the cells were collected and the samples were prepared according to the manufacturer’s instructions. The absorbance was detected at 565 nm by using infinite M1000 PRO multiscan spectrum (Tecan, Switzerland).

### Luciferase Assay

1×10^4^ BV2 cells were seeded in 24-well plates, and transfected with lentivirus NF-κB and AP-1 reporters (QIAGEN, Hilden, Japan) for 24 h and then screened with puromycin (2.5 μg/ml) to obtain BV2 cell lines stably expressing NF-κB and AP-1 reporter elements. Subsequently, BV2 cells stably expressing NF-κB and AP-1 reporter elements were treated with LPS (100 ng/ml) with or without RRx-001 (5 μM). After treatments, the activities of NF-κB and AP-1 promoters were detected with Firefly Luciferase Reporter Gene Assay Kit (Beyotime Biotechnology, Shanghai, China) at 560 nm by using Luminoskan™ Ascent (Thermo Fisher Scientific, Waltham, United States).

### Nucleus-Cytoplasm Fractionation Assay

BV2 cells were pretreated with RRx-001 (5 μM) for 12 h, and then stimulated with LPS (100 ng/ml) for 15 min. After treatments, the cells were collected and lysed in nuclear extracting buffer (0.1 mM EDTA, 0.5 mM phenylmethylsulfonyl fluoride (PMSF), 1 mM DTT (Dithiothreitol), 2 mM MgAc, 3 mM CaCl_2_ and 320 mM sucrose) with 0.5% NP-40. The cell samples were kept on ice for 30 min, and were shaken every 5 min. Subsequently, the supernatant was collected and prepared as a cytoplasmic fraction after centrifugation at 600 *g* at 4°C for 15 min. The precipitate was washed twice with nuclear extracting buffer as the nuclear fraction. Finally, the expression of p65 protein in the cytoplasm and the nucleus was verified by immunoblotting.

### Immunoprecipitation

1×10^5^ BV2 cells cultured in 100 mm cell culture dishes (Thermo Fisher Scientific, Waltham, United States) were treated with LPS (100 ng/ml) for 15 min with or without pretreatment of RRx-001 (5 μM). After treatments, the cells were collected and lysed in IP lysis buffer (150 mM NaCl, 1% NP-40, 1% sodium deoxycholate, and 25 mM Tris-HCl, pH 7.6) with a protease inhibitor cocktail (Roche, Switzerland). After ultrasonic on ice, a certain volume of protein product was taken to prepare samples as an input sample. All remaining products were put into Protein A/G PLUS-Agarose (Santa Cruz Biotechnology, Santa Cruz, United States) loaded with anti-TLR4 antibody and mixed overnight at 4°C. After centrifugation at 3,000 rpm for 5 min, the precipitate was washed with PBS 3 times as an IP sample. The interaction between TLR4 and MyD88 was verified by immunoblotting.

### Immunofluorescence

1×10^4^ BV2 cells were seeded in 24-well plates, and pretreated with RRx-001 (5 μM) and 5×10^4^ primary microglia with RRx-001 (0.8 μM) for 12 h, and then stimulated with LPS (100 ng/ml) for 15 min. After treatments, the cells were fixed with 4% paraformaldehyde for 15 min, and permeabilized with 0.5% Triton X-100 for 10 min. The cells were then blocked with 2.5% BSA (Bovine serum albumin) for 1 h after washing with PBS three times. Subsequently, the cells were incubated with anti-p65 or anti-NLRP3 antibody overnight at 4°C, followed by incubation with secondary antibody for 1 h in the dark. After washing with PBS three times, the cells were stained with DAPI (Beyotime Biotechnology, Shanghai, China) at room temperature for 5 min. Finally, the distribution of p65 protein and the activation of NLRP3 inflammasome within the cell was imaged using LSM 710 Laser Scanning Confocal Microscope (Carl Zeiss, Jena, Germany).

### Immunohistochemistry

C57BL/6 mice were pretreated with RRx-001 to establish an *in vivo* model of neuroinflammation. Seven days after LPS injection, the mice were perfused with precooled PBS and then 4% paraformaldehyde and their brains were taken and immersed in 4% paraformaldehyde for 3 days at 4°C. The brains were then dehydrated at 4°C for 3 days in 20 and 30% sucrose, respectively. After being frozen overnight at −80°C, 20 µM-thick slices of the midbrain were obtained at −20°C by using a CM1950 Freezing Microtome (Leica, Wetzlar, Germany). Microglia and astrocytes were visualized by immunohistochemical staining with anti-Iba1 antibody (Wako Chemicals, Tokyo, Japan) and anti-GFAP antibody (Sigma-Aldrich, St. Louis, United States), respectively. The dopaminergic neurons were shown by immunohistochemical staining with anti-tyrosine hydroxylase (TH) antibody (Millipore, Billerica, United States). The nuclei were stained with DAPI. Finally, the slices were imaged by using an inverted Nikon Eclipse Microscope (Nikon, Tokyo, Japan) and the fluorescence intensity was analyzed by using ImageJ software.

### Statistical Analysis

The results of all parallel experiments were shown as the means ± SEM. One-way ANOVA followed by Bonferroni’s post-hoc test was used for multiple comparisons between groups and the significance of differences between groups was performed with GraphPad Prism 8.0 software (GraphPad Software, San Diego, United States). The difference was statistically significant, at least with *p* < 0.05.

## Results

### RRx-001 Inhibits the Transcriptional Activation and Production of LPS-Induced Pro-Inflammatory Factors in Microglial Cells

**FIGURE 1:**
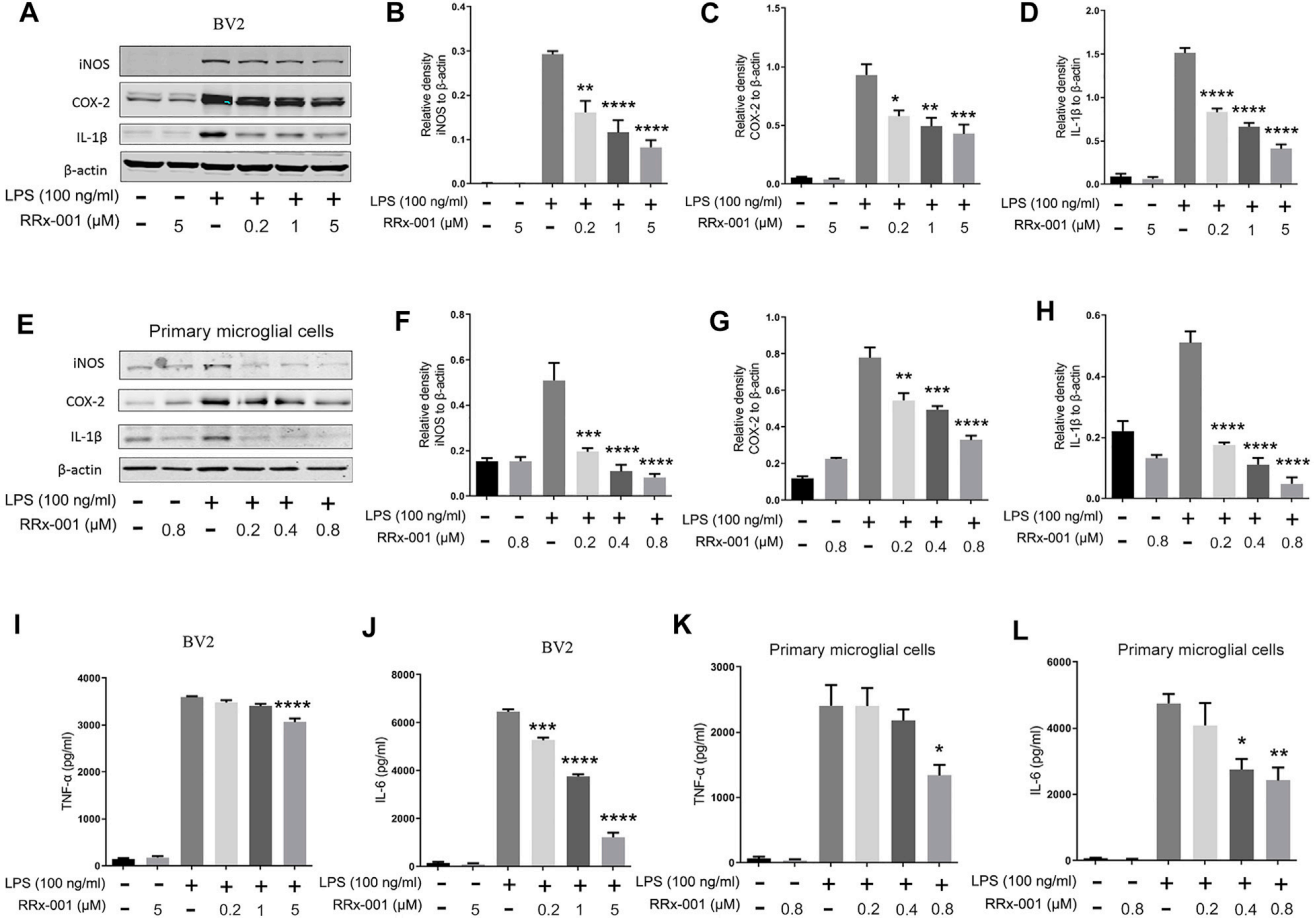
RRx-001 inhibited the production of LPS-induced pro-inflammatory factors. **(A–H)** BV2 cells were pretreated with RRx-001 at proportionate doses of 0.2, 1 and 5 µM and primary microglia with RRx-001 at doses of 0.2, 0.4 and 0.8 µM for 12 h, followed by LPS (100 ng/ml) for 12 h. After the cells were harvested, the protein levels of iNOS, COX-2, IL-1β and β-actin were detected using western blotting assay. The relative intensity of iNOS, COX-2 and IL-1β relative to β-actin was quantified in the bar figures. **(I–L)** BV2 cells were pretreated with RRx-001 at proportionate doses of 0.2, 1 and 5 µM and primary microglia with RRx-001 at doses of 0.2, 0.4 and 0.8 µM for 12 h, followed by LPS (100 ng/ml) for 24 h. After the cultured media were collected, the levels of TNF-α and IL-6 were measured by ELISA kits. The values from three parallel experiments were shown as the means ± SEM. One-way ANOVA followed by Bonferroni’s post-hoc test was used for multiple comparisons between groups, *p < 0.05, **p < 0.01, ***p < 0.001, ****p < 0.0001, compared with those treated with LPS alone.

To explore if suppression of neuroinflammation by RRx-001 is mediated by inhibition of microglia, BV2 microglial cell line and primary microglia were used. Firstly, we investigated the drug toxicity of RRx-001 in these cells. As shown in Supplementary Figures S1A–D, RRx-001 at the doses of 0.2, 1, and 5 µM in BV2 cells or 0.2, 0.4, and 0.8 µM in primary microglial cells had no effect on cell viability and morphology with or without LPS treatment. Then we investigated the inhibitory effect of RRx-001 at three different concentrations on microglial activation in these 2 cells. Upon LPS stimulation, RRx-001 pretreatment significantly reduced the protein levels of iNOS, COX-2 and IL-1β in a dose-dependent manner (Figures 1A–H). Furthermore, the release of pro-inflammatory cytokines tumor necrosis factor (TNF)-α and interleukin (IL)-6 in the culture media after LPS stimulation was significantly diminished by RRx-001 pretreatment (Figures 1I–L).

**FIGURE 2:**
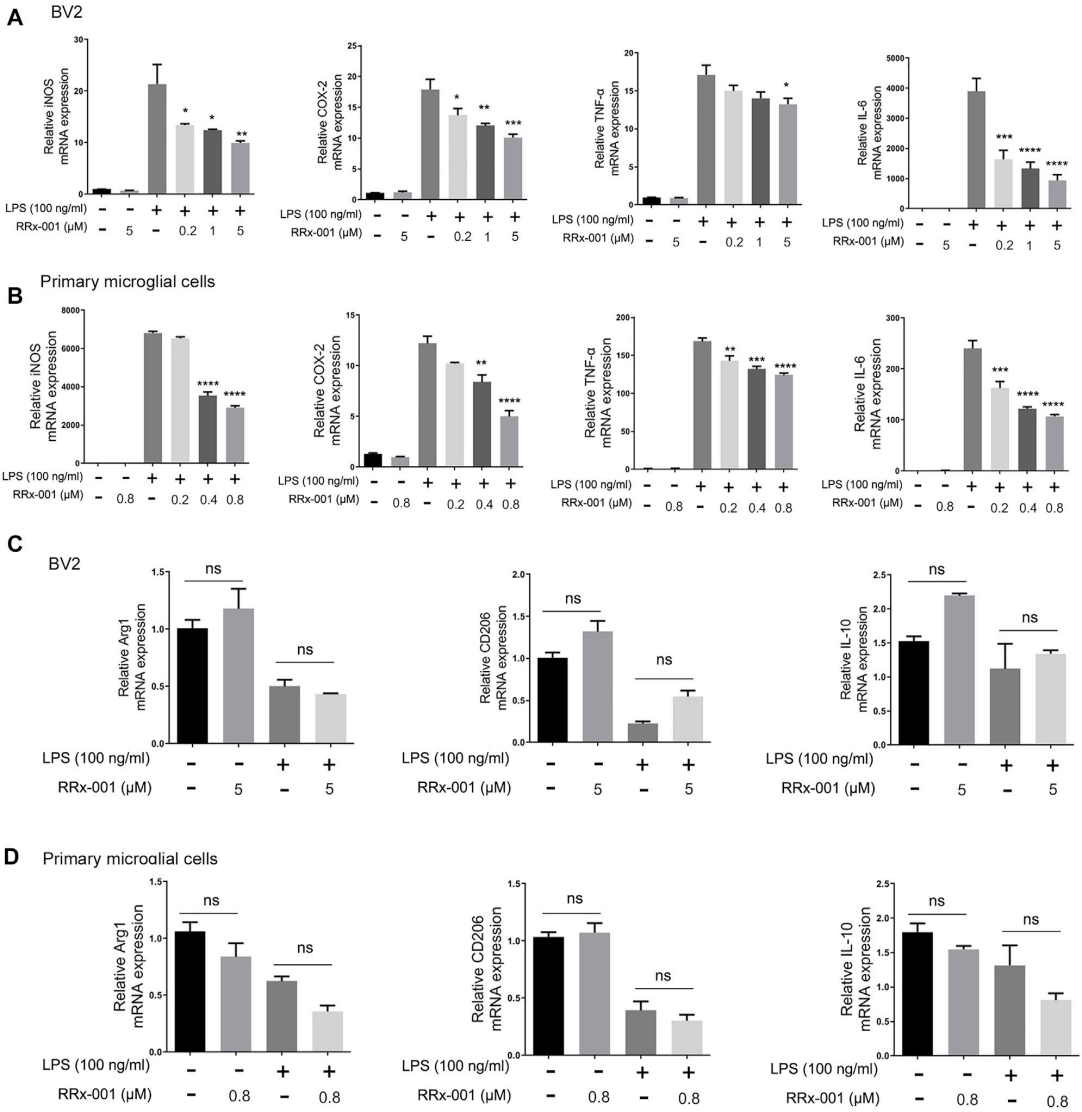
RRx-001 inhibited the transcriptional activation of LPS-induced proinflammatory cytokines in BV2 cells and primary microglia. **(A,B)** BV2 cells were pretreated with RRx-001 at proportionate doses of 0.2, 1 and 5 µM and primary microglia with RRx-001 at doses of 0.2, 0.4 and 0.8 µM for 12 h and followed by LPS (100 ng/ml) stimulation for 6 h. After the total RNA was extracted, the mRNA levels of iNOS, COX-2, TNF-α and IL-6 were determined with qRT-PCR. The data from three independent experiments were shown as the means ± SEM. One-way ANOVA followed by Bonferroni’s post-hoc test was used for multiple comparisons between groups, *p < 0.05, **p < 0.01, ***p < 0.001, ****p < 0.0001, compared with those treated with LPS alone. **(C,D)** The mRNA levels of Arg1, IL-10 and CD206 in BV2 cells and primary microglia treated as described in a and b were determined with qRT-PCR. The data from three independent experiments were shown as the means ± SEM. ns, not significantly different.

Next, we investigated if LPS-induced proinflammatory cytokines were affected by RRx-001 at the transcription level. The mRNA levels of the proinflammatory cytokines iNOS, COX-2, IL-6, and TNF-α were significantly increased in LPS-treated BV2 cells and primary microglial cells, while the pretreatment with RRx-001 significantly reduced the mRNA levels of pro-inflammatory factors in a dose-dependent manner (Figures 2A,B). After activation, microglia *in vivo* have two opposite states, which can be divided into the pro-inflammatory phenotype (M1) and the anti-inflammatory phenotype (M2), expressing the aforementioned pro-inflammatory factors, such as iNOS, COX-2, IL-6 and TNF-α, and anti-inflammatory factors, such as Arg1, IL-10 and CD206 (Hu et al., 2014), respectively. However, RRx-001 pretreatment did not affect the expression of anti-inflammatory factors (Figures 2C,D). This suggests that RRx-001 may specifically inhibit the activation of proinflammatory microglia cells.

### RRx-001 did Not Inhibit the Expression and Activity of G6PD in BV2 Cells

Subsequently, we would like to further investigate the underlying mechanism of RRx-001s effects in the anti-inflammation. It has been reported that the anti-inflammatory effect of RRx-001 may be related to the inhibition of glucose -6-phosphate dehydrogenase (G6PD). Therefore, we detected the expression and activity of G6PD in BV2 cells treated with RRx-001. However, there was no significant change in the expression of G6PD and the concentration of intracellular NADPH after treatment with LPS and RRx-001 following the experimental drug concentration (Supplementary Figures S2A–D).

### RRx-001 Inhibits LPS-Induced Activation of NF-кB and MAPK Signaling Pathways in Microglial Cells

**FIGURE 3:**
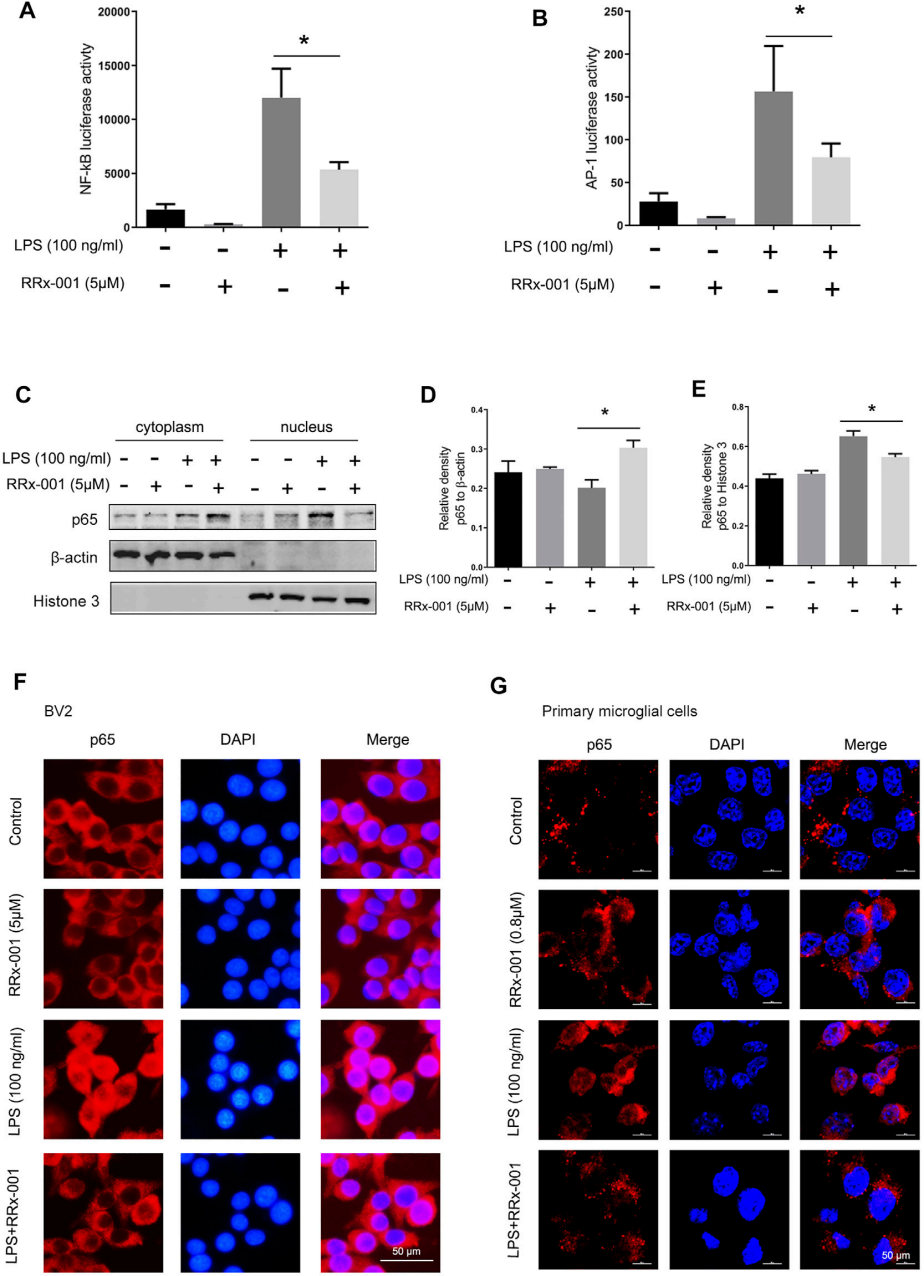
RRx-001 inhibited LPS-induced the activation of transcription factors NF-кB and AP-1 in BV2 cells and primary microglia. **(A,B)** The cells were treated in the same way as in Figure 2 After the cells were harvested, the transcriptional expression of NF-κB and AP-1 was measured with a luciferase assay. **(C–E)** BV2 cells were pretreated with RRx-001 (5 µM) for 12 h and then stimulated with LPS (100 ng/ml) for 15 min. The levels of p65 protein in the cytoplasm and nucleus were determined by subcellular fractionation methods. And the relative density of p65 relative to cytoplasmic marker (β-actin) and nuclear marker (histone 3) was quantitatively analyzed in the following panels. **(F,G)** The localization of p65 protein in BV2 cells and primary microglia treated as described in **(C)** was stained with immunofluorescence, and the nuclei were stained with DAPI. The cells were then imaged with fluorescence microscopy. Scale bar, 50 µm. The values from three independent experiments were shown as the means ± SEM. One-way ANOVA followed by Bonferroni’s post-hoc test was used for multiple comparisons between groups, *p < 0.05, compared with those treated with LPS alone.

The transcription factors nuclear factor κB (NF-κB) and activator protein-1 (AP-1) translocate from cytoplasm to nucleus to regulate the transcription and expression of proinflammatory cytokines in LPS-activated BV2 cells (Anrather et al., 2006; Huo et al., 2011; Gay et al., 2014). Thus we investigated if RRx-001 inhibits the expression of proinflammatory cytokines by affecting these two transcriptional factors. The transcriptional activity of NF-κB and AP-1 was detected by luciferase reporter gene assays which were constructed in BV2 cells stably expressing NF-κB and AP-1 reporter elements. After LPS stimulation, the transcriptional activity of NF-κB and AP-1 was markedly increased, while RRx-001 pretreatment reduced its upregulation (Figures 3A,B). In addition, the distribution of the NF-κB p65 subunit was measured by the nucleus-cytoplasm fractionation assay (Figures 3C–E) and the immunofluorescence staining (Figures 3F,G). Upon LPS stimulation, the level of p65 in the nucleus was observably increased, while RRx-001 pretreatment could significantly reduce the nuclear NF-κB p65 subunit in BV2 cells and primary microglia cells.

**FIGURE 4:**
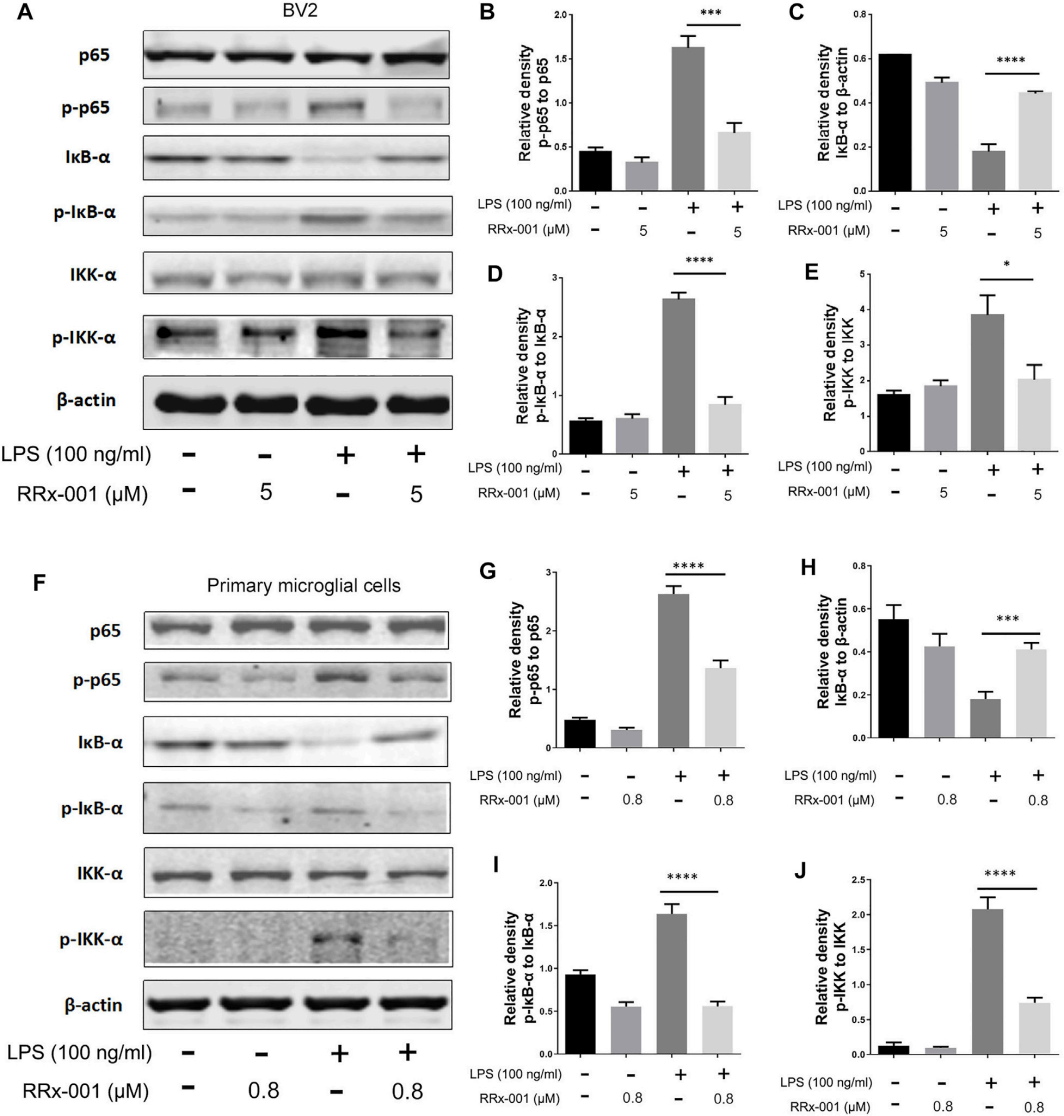
RRx-001 inhibited LPS-induced the activation of NF-кB signaling pathway. **(A–J)** BV2 cells were pretreated with RRx-001 (5 µM) and primary microglia with RRx-001 (0.8 µM) for 12 h, followed by LPS (100 ng/ml) for 15 min. After the cells were harvested, the protein levels of p65, p-p65, IκB-α, p-IκB-α, IKK-α, p-IKK-α and β-actin were detected using western blotting assay. The relative intensity of p-p65, IκB-α, p-IκB-α and p-IKK-α, respectively relative to p65, β-actin, IκB-α and IKK-α was quantified in the bar figures. The values from three independent experiments were shown as the means ± SEM. One-way ANOVA followed by Bonferroni’s post-hoc test was used for multiple comparisons between groups, *p < 0.05, **p < 0.01, ****p < 0.0001, compared with those treated with LPS alone.

The transcription factor nuclear factor κB (NF-κB) mediates the initiation and transcription of pro-inflammatory factors in LPS-induced inflammation (Hayden and Ghosh, 2008). Under normal conditions, NF-κB is sequestered in the cytoplasm due to its binding to inhibiting protein IκBs. At the onset of inflammation, the two subunits of NF-κB, p65, and p50, are dissociated from the inhibitory protein IκBs, which is activated by phosphorylated protein IkB kinase (IKK) and degraded by ubiquitination, while the phosphorylated p65 is translocated into the nucleus and initiates the transcription of pro-inflammatory factors (Hu et al., 2012). The degradation of IκB-α and the activation of phosphorylated protein p-p65, p-IκB-α, and p-IKK-α were induced by LPS in BV2 cells and primary microglia cells, which were dramatically suppressed by pretreatment of RRx-001 (Figures 4A–J).

**FIGURE 5:**
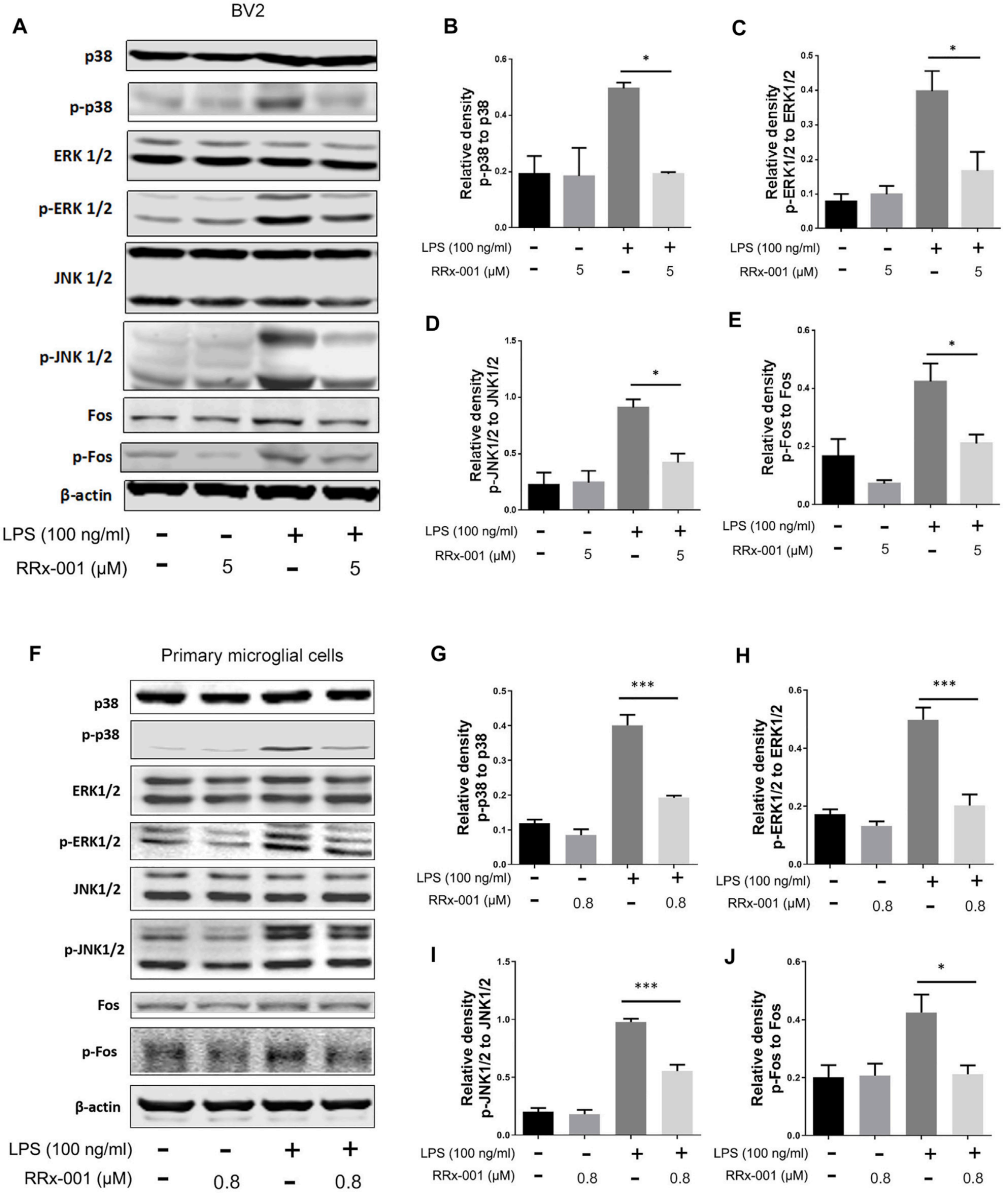
RRx-001 inhibited LPS-induced the activation of MAPK signaling pathway. **(A–J)** The cells were treated in the same way as in Figure 4. After the cells were harvested, the protein levels of p38, p-p38, ERK1/2, p-ERK1/2, JNK1/2, p-JNK1/2, Fos, p-Fos and β-actin were detected using western blotting assay. The relative intensity of p-p38, p-ERK1/2, p-JNK1/2 and p-Fos, respectively relative to p38, ERK1/2, JNK1/2 and Fos was quantified in the bar figures. The values from three independent experiments were shown as the means ± SEM. One-way ANOVA followed by Bonferroni’s post-hoc test was used for multiple comparisons between groups, *p < 0.05, **p < 0.01, ***p < 0.001, ****p < 0.0001, compared with those treated with LPS alone.

In LPS-stimulated inflammation, activation of mitogen-activated protein kinase (MAPK) signaling pathways characterized by phosphorylation of p38 MAPK, extracellular signal-regulated kinase (ERK1/2) and c-jun NH2-terminal kinase (JNK1/2) is involved in the transcriptional expression of inflammatory factors. Subsequent activation of MAP kinases results in the formation of an AP-1 transcription factor by activating c-Jun, which translocates into the nucleus and binds with Jun or Fos family members (Schonthaler et al., 2011). Phosphorylation and activation of proteins p38 MAPK, ERK1/2, JNK1/2, and Fos were stimulated by LPS in BV2 cells and primary microglia cells, which were markedly decreased by RRx-001 pretreatment (Figures 5A–J).

### RRx-001 Inhibits the Expression and Activation of NLRP3 Inflammasomes Induced by LPS and ATP in Microglial Cells

**FIGURE 6:**
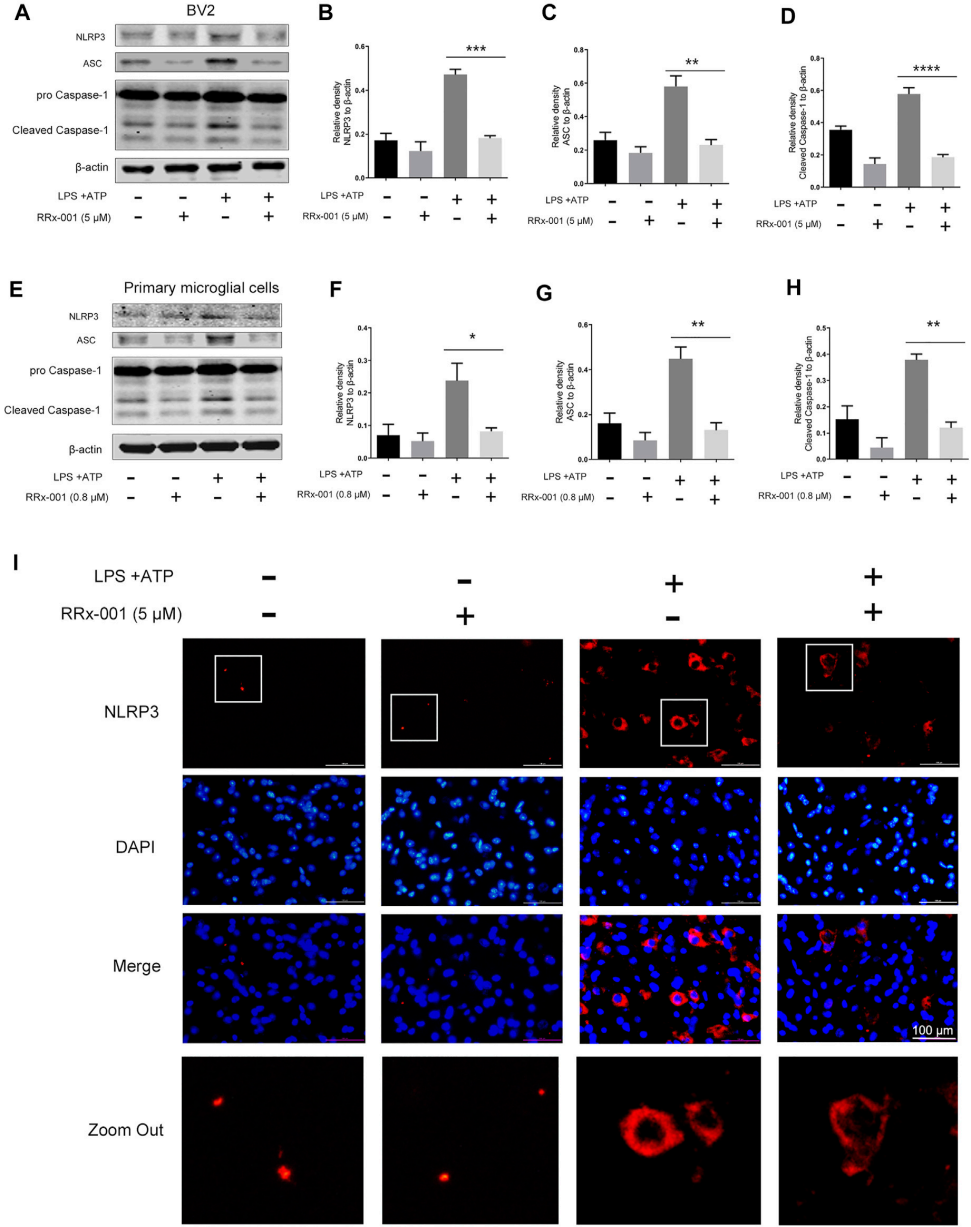
RRx-001 inhibited the upregulation of NLRP3 inflammasome induced by LPS and ATP in BV2 cells and primary microglia. **(A–H)** BV2 cells were pretreated with RRx-001 (5 µM) and primary microglia with RRx-001 (0.8 µM) for 12 h, followed by LPS (100 ng/ml) for 12 h and then stimulated with ATP (1 mM) for another 2 h. After the cells were harvested, the protein levels of NLRP3, ASC, caspase 1 and β-actin were detected using a western blotting assay. The relative intensity of NLRP3, ASC and cleaved-caspase 1 relative to β-actin was quantified in the bar figures. The values from three independent experiments were shown as the means ± SEM. One-way ANOVA followed by Bonferroni’s post-hoc test was used for multiple comparisons between groups, **p < 0.01, ***p < 0.001, ****p < 0.0001, compared with those treated with LPS alone. **(I)** The expression of NLRP3 in BV2 cells treated as described in a was stained with immunofluorescence, and the nuclei were stained with DAPI. The cells were then imaged with fluorescence microscopy. Scale bar, 100 µm.

NF-κB activation not only promotes the transcription of pro-inflammatory factors such as pro-IL-1β but also positively regulates the expression of the NLR family pyrin domain containing 3 (NLRP3) (Bauernfeind et al., 2009). After induction, NLRP3 is combined with apoptosis-associated speck-like protein containing a C-terminal caspase recruitment domain (ASC) to assemble inflammasome complexes, triggering cleavage of dormant procaspase-1 into active caspase-1, thereby converting the cytokine precursor pro-IL-1β into mature biologically active IL-1β (He et al., 2016). Therefore, we investigated whether RRx-001 could suppress the expression of NLRP3 inflammasome beyond inhibiting NF-κB activation. The increased protein levels of NLRP3, ASC, and caspase 1 after stimulation by LPS and ATP were effectively reduced by pretreatment of RRx-001 in BV2 cells and primary microglial cells (Figures 6A–H). In addition, the immunofluorescence assay also showed that RRx-001 inhibited the upregulation of NLRP3 inflammasome induced by LPS and ATP (Figure 6I).

### RRx-001 Inhibits the Nox-Derived Oxidative Stress Responses Induced by LPS in Microglial Cells

**FIGURE 7:**
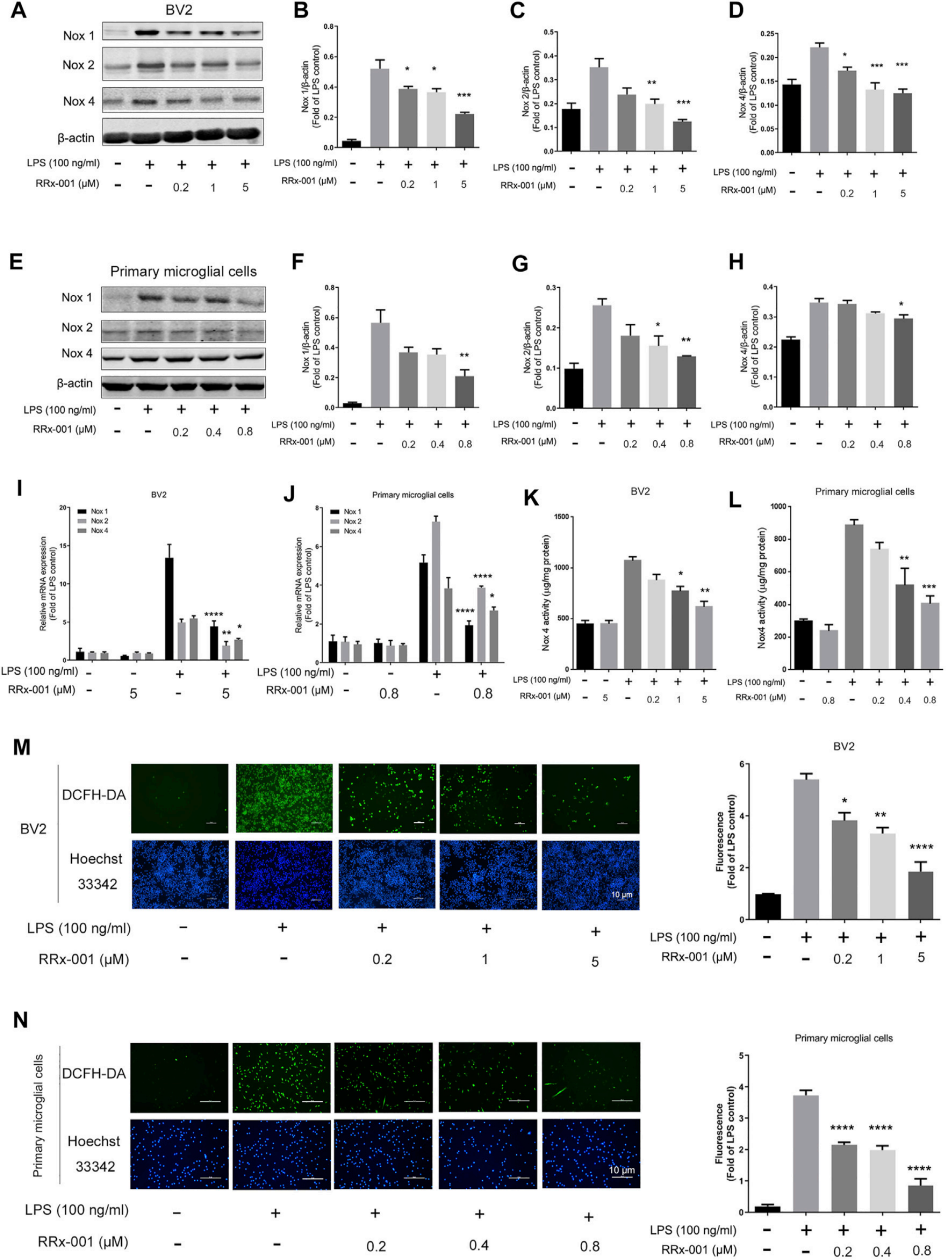
RRx-001 ameliorated LPS-induced oxidative stress by inhibiting the Nox family. **(A–H)** The cells were treated in the same way as in Figure 1 **(A–H)**. After the cells were harvested, the protein levels of Nox1, Nox2, Nox4 and β-actin were detected using a western blotting assay. The relative intensity of Nox4 relative to β-actin was quantified in the bar figures. **(I,J)** The cells were treated in the same way as in Figures 2A,B. After the total RNA was extracted, the mRNA levels of Nox1, Nox2 and Nox4 were determined with qRT-PCR. **(K,L)** The cells were treated in the same way as in Figures 1I,J. After the cells were harvested, the activity of Nox4 was measured by ELISA kits. **(M,N)** BV2 cells were pretreated with RRx-001 at proportionate doses of 0.2, 1 and 5 μM, and primary microglia with RRx-001 at doses of 0.2, 0.4 and 0.8 µM for 12 h, and then stimulated with LPS (100 ng/ml) for 30 min. The intracellular levels of ROS were measured by using the fluorescent probe DCFH-DA (10 μmol/L), and the nuclei were stained with Hoechst. The cells were then imaged with fluorescence microscopy, and the quantification of fluorescence intensity is shown in the bar figure. Scale bar, 10 µm. The values from three independent experiments were shown as the means ± SEM. One-way ANOVA followed by Bonferroni’s post-hoc test was used for multiple comparisons between groups, *p < 0.05, **p < 0.01, ***p < 0.001, ****p < 0.0001, compared with those treated with LPS alone.

Neuroinflammation is often accompanied by the increase of superoxides and ROS, and the NADPH oxidase family, which is the main source of intracellular ROS, especially Nox1, Nox2, and Nox4, is transcribed by NF-κB and AP-1-dependent regulation (Anrather et al., 2006; Huo et al., 2011). Therefore, we investigated if RRx-001 could suppress the expression of Nox isoforms and ROS production. The LPS-induced increase in Nox1, Nox2, and Nox4 protein levels were reduced by RRx-001 pretreatment in BV2 cells and primary microglial cells (Figures 7A–H). Meanwhile, the mRNA levels in BV2 cells and primary microglial cells also displayed a similar trend (Figures 7I,J). In addition, the increased activity of Nox4 upon LPS stimulation was inhibited after RRx-001 pretreatment in a dose-dependent manner (Figures 7K,L). Furthermore, the immunofluorescence assay more intuitively indicated that RRx-001 could reduce the generation of LPS-induced ROS in a dose-dependent manner (Figures 7M,N).

### RRx-001 Inhibits LPS-Induced Activation of TAK1, But Did Not Block MyD88 Recruitment to TLR4

**FIGURE 8:**
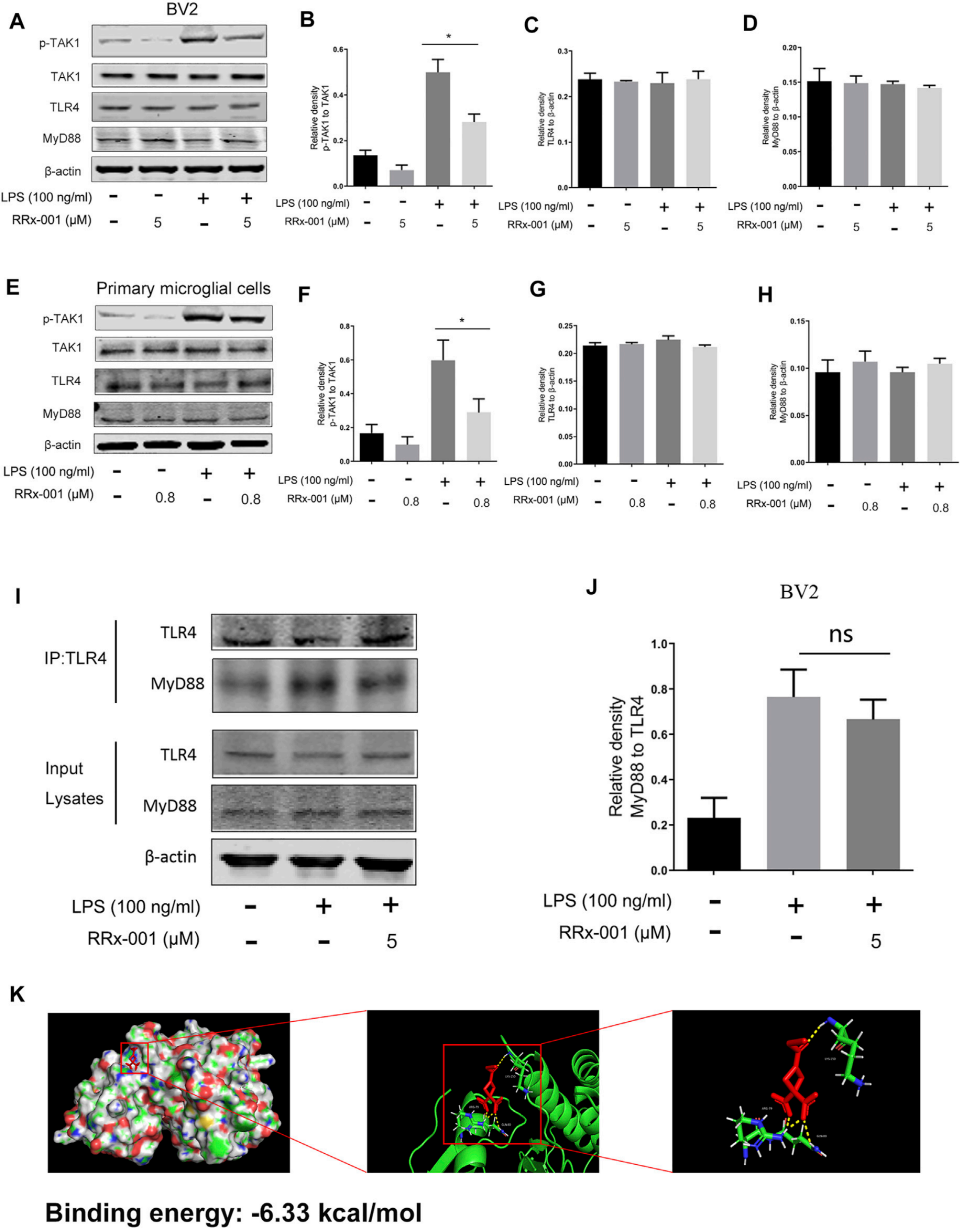
RRx-001 inhibited LPS-induced activation of TAK1, but did not block MyD88 recruitment to TLR4. **(A–H)** The cells were treated in the same way as in Figure 4. After the cells were harvested, the protein levels of TLR4, MyD88, TAK1, p-TAK1, and β-actin were detected using western blotting assay. The relative intensity of p-TAK1, TLR4, and MyD88, respectively relative to TAK1 and β-actin was quantified in the bar figures. **(I,J)** The cells were treated in the same way as in Figure 3C. After the cells were harvested, the proteins were immunoprecipitated with TLR4 using magnetic beads. Immunocomplexes were determined by western blotting assay with anti-TLR4 and anti-MyD88 antibodies, and the relative intensity of MyD88 relative to TLR4 was quantified. **(K)** The interaction between RRx-001 and TAK1 was analyzed by molecular docking. The values from three independent experiments were shown as the means ± SEM. One-way ANOVA followed by Bonferroni’s post-hoc test was used for multiple comparisons between groups, *p < 0.05, compared with those treated with LPS alone.

TLR4, as a transmembrane receptor expressed on the microglia membrane, plays a key role in the regulation of neuroinflammation (Campolo et al., 2019). After stimulation by LPS, the dimerization of TLR4 recruits myeloid differentiation factor 88 (MyD88) and TIR-domain-containing adaptor molecules (TIRAP) to activate the downstream protein TGF β-activated kinase 1 (TAK1), which subsequently mediates the synthesis and release of pro-inflammatory factors through activation of downstream signaling pathways, including NF-κB and MAPK signaling pathways (Campolo et al., 2019). Therefore, we examined the activation of TAK1 phosphorylation in BV2 cells and primary microglia cells. RRx-001 pretreatment significantly inhibited LPS-induced TAK1 phosphorylation without a significant effect on the protein levels of TLR4 and MyD88 (Figures 8A–H). Furthermore, the immunoprecipitation assay showed that MyD88 is recruited to the TLR4 upon LPS stimulation, which was not blocked by pretreatment with RRx-001 (Figures 8I,J). And, the molecular docking analysis of RRx-001 and TAK1 further confirmed that RRx-001 could strongly bind to TAK1 at multiple amino acid aggregation sites, including ARG-79, GLN-80, and LYS-150 (Figure 8K). These data suggest that RRx-001 has an inhibitory effect on TAK1 activation.

### RRx-001 Inhibits Neuronal Cell Death Mediated by Neuroinflammation

**FIGURE 9:**
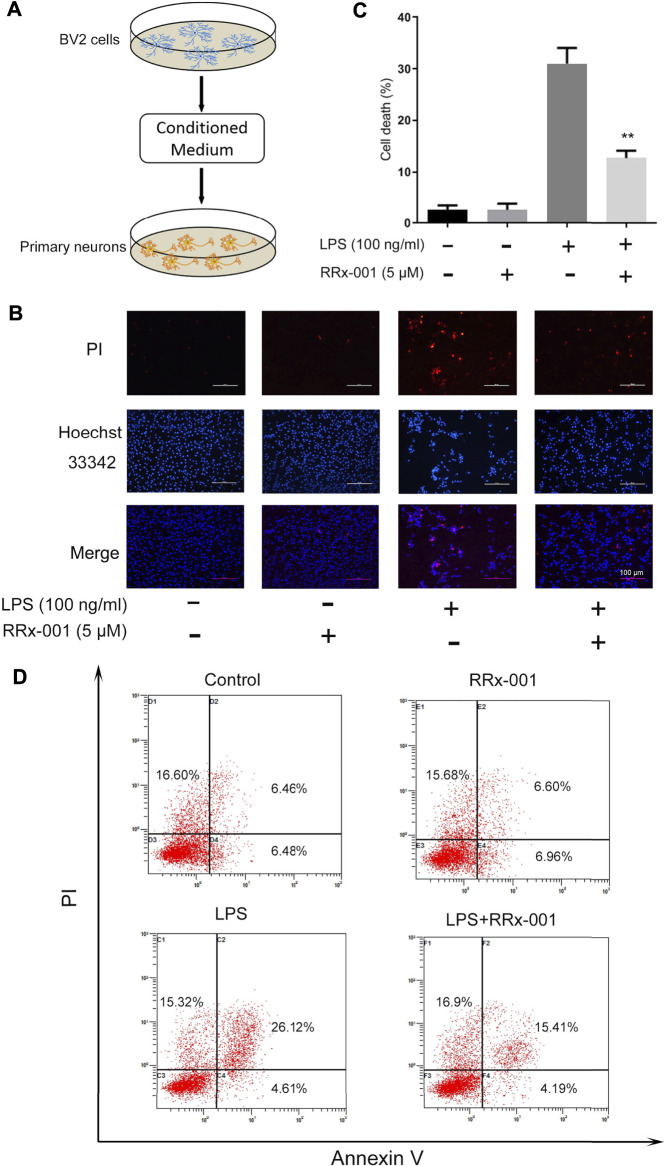
RRx-001 inhibited neuronal cell death mediated by neuroinflammation. **(A)** The scheme of the conditioned medium (CM) assay. BV2 cells were pretreated with RRx-001 (5 µM) for 12 h and then stimulated with LPS (100 ng/ml) for 24 h. The media from different groups were collected and used for the culture of primary neurons. **(B,C)** After being cultured in the conditional medium for 24 h, cell apoptosis was detected with propidium iodide (PI), and the nuclei were stained with Hoechst. The cells were then imaged with fluorescence microscopy, and the quantification of cell death is shown in the bar figure. Scale bar, 100 µm. **(D)** BV2 cells were treated as (A) and subjected to flow cytometry analysis. The values from three independent experiments were shown as the means ± SEM. One-way ANOVA followed by Bonferroni’s post-hoc test was used for multiple comparisons between groups, **p < 0.01, compared with those treated with LPS alone.

A large number of studies have shown that inflammatory cytokines released by activated microglia directly lead to neuronal death (Gu et al., 2018). Therefore, we hypothesized that RRx-001 could inhibit inflammation-induced neuronal death. We verified it with a conditioned medium (CM) assay (Figure 9A). The supernatant medium was collected from LPS-treated BV2 cells with or without RRx-001 and was used for the culture of primary neurons. Finally, cell apoptosis was detected through Hoechst and propidium iodide (PI) staining. The apoptotic rate of primary neurons cultured with LPS-primed BV2 cell conditioned medium was significantly increased, while the cell death of neurons cultured with RRx-001 pretreated, LPS-primed BV2 cell conditioned medium was reduced (Figures 9B,C). Similarly, similar results were found by flow cytometry (Figure 9D). RRx-001 decreased the toxicity of CM from LPS-stimulated BV2 cells.

In addition, we considered whether RRx-001 might affect cell viability in the central nervous system while inhibiting microglia-activated inflammation. Subsequently, we investigated the effects of RRx-001 at the doses of 0.2, 0.4, 0.8, 1.6 and 3.2 µM on cell viability and morphology in primary astrocytes and primary neurons. As shown in Supplementary Figures S1E–G, RRx-001 did not affect cell viability and morphology at various doses in primary astrocytes, whereas primary neurons were damaged at a dose of 1.6 µM.

### RRx-001 Suppresses LPS-Stimulated Microglia Activation and Loss of Dopaminergic Neurons in the Midbrain

**FIGURE 10:**
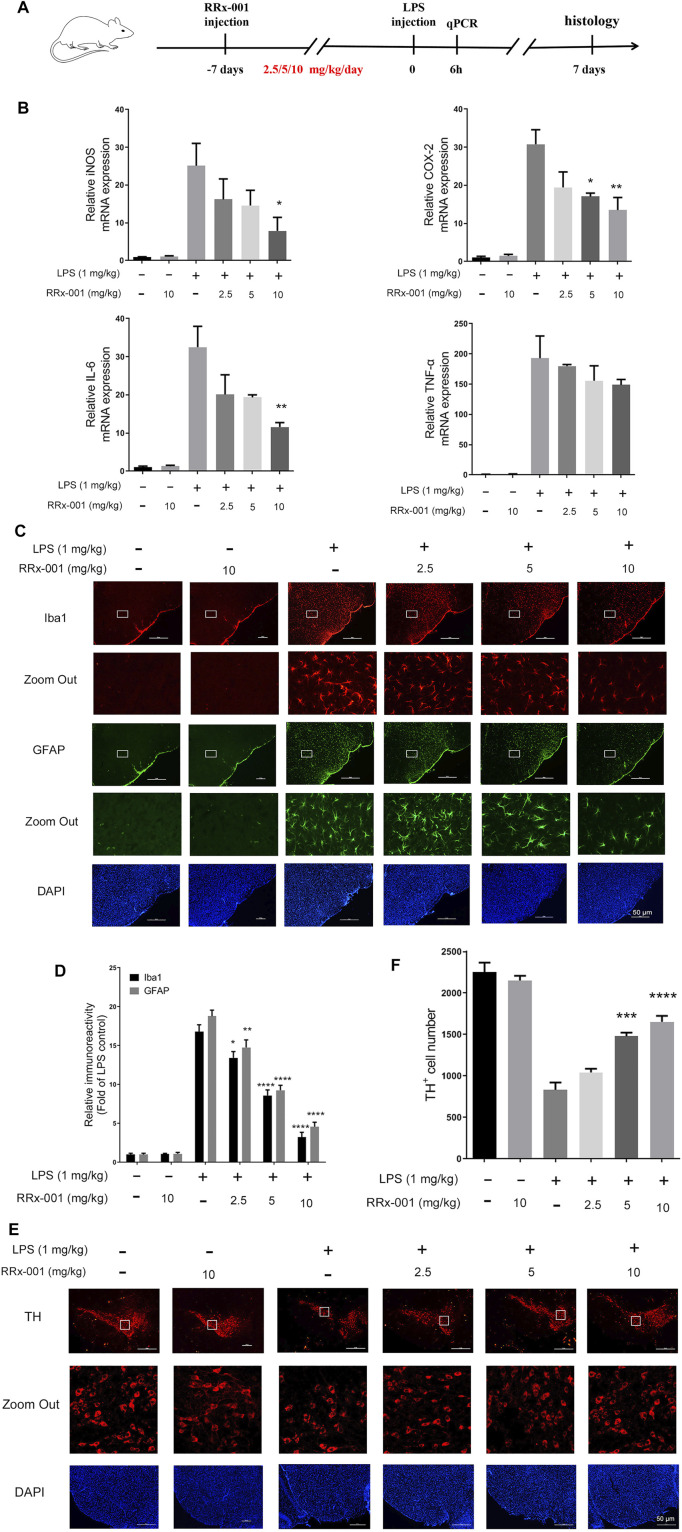
RRx-001 suppressed LPS-induced neuroinflammation and dopaminergic (DA) neuronal loss in the midbrain. **(A)** The scheme for *in vivo* experiments in animals. RRx-001 with different doses (2.5, 5, 10 mg/kg) was injected intraperitoneally once a day for 7 days before stereotactic injection of LPS. Six hours after modeling, the total RNA was extracted from the midbrain for real-time quantitative PCR assay. Seven days after LPS injection, the mice were sacrificed, and 20 µM-thick slices of the midbrain were sectioned by using a freezing microtome for immunohistochemical assay. **(B)** After the total RNA was extracted, the mRNA levels of iNOS, COX-2, TNF-α, and IL-6 were determined with qRT-PCR. **(C,D)** Microglia and astrocytes were shown by immunohistochemical staining with anti-Iba1 and anti-GFAP antibodies, respectively. And the nuclei were stained with DAPI. The microglia and astrocytes were then imaged with fluorescence microscopy, and the quantification of the relative fluorescence intensity is shown in the bar figure. **(E,F)** The dopaminergic neurons were shown by immunohistochemical staining with anti-tyrosine hydroxylase (TH) antibody, and the nuclei were stained with DAPI. TH^+^ neurons were then imaged with fluorescence microscopy, and the quantification of the relative fluorescence intensity was shown in the bar figure. Scale bar, 50 µm. The values from six mice of each group were shown as the means ± SEM. One-way ANOVA followed by Bonferroni’s post-hoc test was used for multiple comparisons between groups, *p < 0.05, **p < 0.01, ***p < 0.001, ****p < 0.0001, compared with those treated with LPS alone.

To investigate the potential role of RRx-001 in neuroinflammation, LPS was injected into the substantia nigra pars compacta (SNpc) of the mouse to establish the inflammation model of the central nervous system (Gu et al., 2018). RRx-001 (2.5, 5, 10 mg/kg) was injected intraperitoneally once a day for 7 days before LPS injection. Stereotactic injection of LPS was performed on the eighth day and no RRx-001 treatment thereafter. The total RNA from mouse midbrains was extracted 6 h after the LPS injection for real-time quantitative PCR assay. After modeling for 7 days, the brains were perfused and were taken for the immunohistochemical assay (Figure 10A). LPS-induced increases in proinflammatory factors in the midbrain of mice, including iNOS, COX-2, IL-6, and TNF-α, were significantly reduced by pre-administration of RRx-001 in a dose-dependent manner (Figure 10B). Subsequently, immunofluorescence staining was applied with antibodies against Iba1 (a microglia marker), GFAP (an astrocyte marker), and TH (a DA neuron marker). Upon LPS administration, the number of Iba1-and GFAP-positive cells in the substantia nigra pars compacta (SNpc) was significantly increased. As expected, the number of positive cells was reduced to varying degrees in the groups of RRx-001 pre-administration (Figures 10C,D). Furthermore, the number of TH^+^ neurons in SNpc reduced in LPS-treated mice and RRx-001 administration rescued dopaminergic neurons in the SNpc (Figures 10E,F).

## Discussion

**FIGURE 11:**
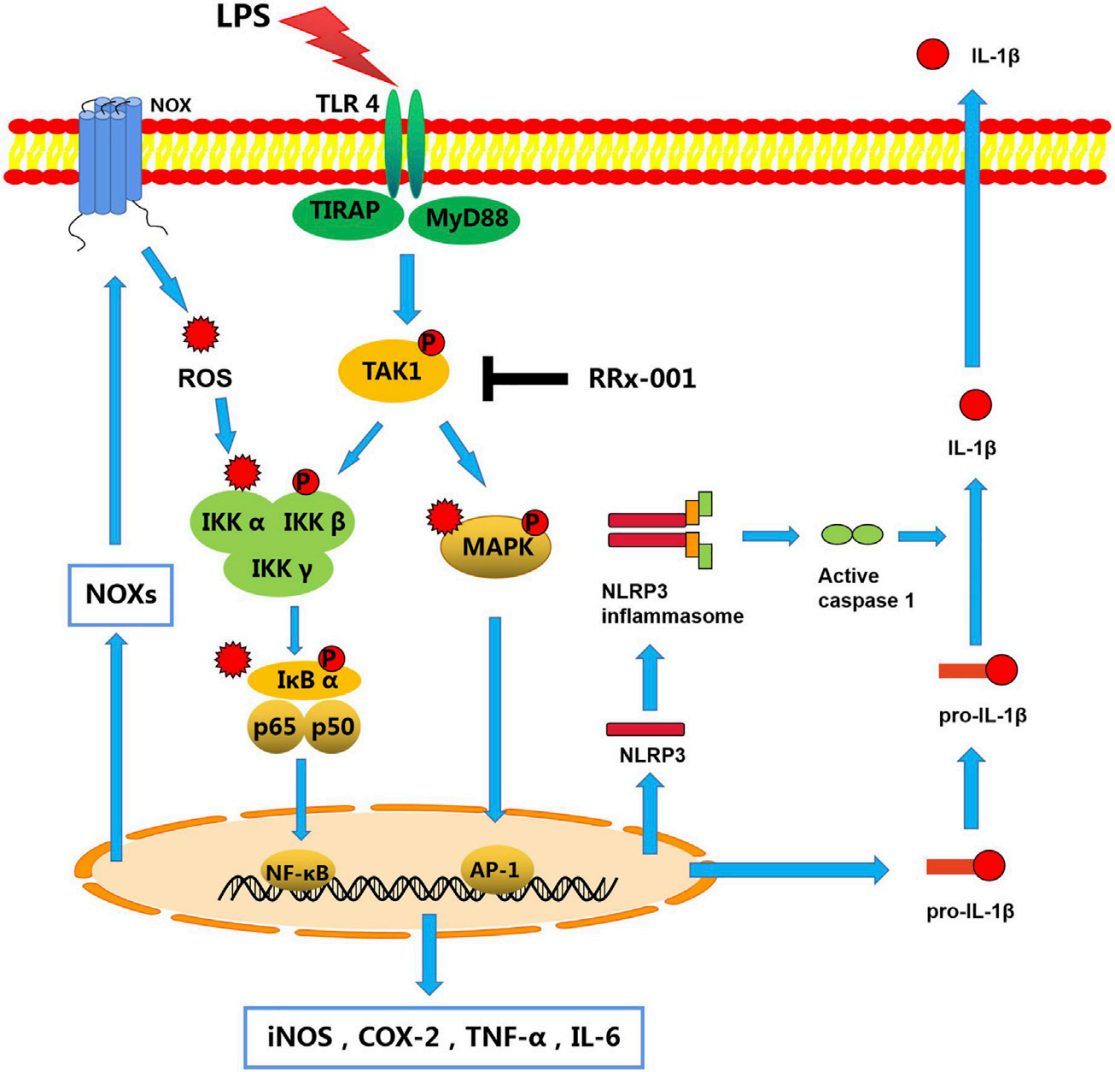
A schematic diagram of RRx-001 mediated improvement of neuroinflammation. In LPS-stimulated microglia, activated TLR4 recruits MyD88 and TIRAP to phosphorylate TAK1, which subsequently promotes the synthesis and release of pro-inflammatory cytokines by activating NF-κB and MAPK signaling pathways. Moreover, the transcriptional expression of the NLRP3 inflammasome is also increased to activate caspase-1, which leads to the maturation and release of IL-1β. In addition, the transcription and expression of Nox isoforms are mediated by transcription factors NF-κB and AP-1 to induce oxidative stress, which exacerbates inflammation through activation of these two pathways. However, RRx-001 pretreatment inhibits the activation of TAK1, resulting in the inhibition of pro-inflammatory cytokines expression, NLRP3 inflammasome activation, and Nox-mediated oxidative stress through the NF-κB and MAPK signaling pathways.

RRx-001, also known as 1-bromoacetyl-3,3-dinitroazetidine (ABDNAZ), is a new clinical-stage chemotherapeutic agent and radiation sensitizer (Oronsky et al., 2016). The phase I trial (NCT01359982) has been completed, and three main phase II trials are currently ongoing, including NCT02489903, NCT02215512, NCT02096354 (Oronsky et al., 2017). It has shown a good effect of killing tumor tissue when combined with radiation therapy in hypoxic tumor cells. Studies have shown that RRx-001 also acts as an epigenetic drug, and its anticancer effect is related to its inhibition of DNA methyltransferases (DNMTs), which induces DNA damage in tumor cells (Li et al., 2017). Furthermore, it has been recently reported that RRx-001 ameliorates systemic inflammation by inhibiting the assembly of NLRP3 inflammasome in mice with colitis and experimental autoimmune encephalomyelitis (EAE) (Chen et al., 2021). This further confirms the important role of RRx-001 in alleviating inflammation. Here, *in vivo* and *in vitro*, RRx-001 was found to mitigate neuroinflammation, and to exert a protective effect on dopaminergic neurons through effectively inhibiting the phosphorylation of TAK1 to lead to the inhibition of NF-κB and MAPK signaling pathways, thereby inhibiting the expression of NLRP3 inflammasome and Nox-derived oxidative stress (Figure 11).

Activation of microglia will not only cause damage to the central nervous system but also affect the occurrence and development of glioma (Altieri et al., 2021). As the most common inducer of the inflammatory response, LPS interacts with TLRs on the cell surface to induce dimerization of TLR4 on the cell membrane. The intracellular region of the TLR4 dimer recruits MAL-MyD88 and TRAM-TRIF, which activates the phosphorylation of TAK1 in a MyD88-dependent pathway, and subsequently activates pro-inflammatory factor production and the transcription of NLRP3 inflammasome through NF-κB and MAPK signaling pathways (Gay et al., 2014). More interestingly, in our study, we found that RRx-001 exhibited an effective inhibitory effect on classical inflammatory signaling pathways by inhibiting TAK1 phosphorylation and transcriptional activity of NF-κB and AP-1, rather than blocking the recruitment of MyD88 to TLR4.

A large number of studies have shown that proinflammatory cytokines transcribed by NF-κB and AP-1 can induce neuronal death, such as IL-1β, TNF-α (Gu et al., 2018). The conditioned medium assay in Figure 9 confirmed that apoptosis of primary neurons cultured with LPS-treated BV2 cell conditioned medium was increased, this effect was attenuated if BV2 cells were pretreated with RRx-001. However, it has not been further established which pro-inflammatory factor is responsible for neuronal death in the present study. To further test the anti-inflammatory and neuroprotective effects of RRx-001 *in vivo*, the present study produced mouse neuroinflammation with LPS. The results showed that preadministration of RRx-001 inhibited microglia activation and neuroinflammation induced by LPS. Moreover, RRx-001 reduced LPS-induced loss of dopamine neurons in midbrain pars compacta, suggesting that RRx-001 could be an effective therapy for some brain diseases where neuroinflammation is implicated.

It has previously been reported that RRx-001 is a G6PD inhibitor (Yu et al., 2019). However, in the present study, we did not find RRx-001s effects on the enzyme levels or activity (measured NADPH levels) in the dose range we used. On the other hand, we found that RRx-001 had an anti-oxidation property. Oxidative stress can be a cause or result of inflammation (Barron et al., 2017). ROS have been reported to activate a variety of molecules to exacerbate inflammation, such as IKKs (Kamata et al., 2002), IκBα (Schoonbroodt et al., 2000), and MAPKs (Torres, 2003). More and more evidence also shows that the high expression of Nox family members in neuroinflammation directly leads to the abnormal increase of ROS, and Nox1, Nox2 and Nox4 are reported to be mainly responsible for the induction of oxidative stress in the central nervous system (Marrali et al., 2018). And, the transcription and expression of Nox isoforms are also regulated by the transcription factors NF-κB and AP-1 (Anrather et al., 2006; Huo et al., 2011). In our study, it was also detected that the expression levels of Nox1, Nox2, and Nox4 in microglia were significantly increased under the stimulation of LPS, which subsequently induced the production of ROS, indicating that the level of ROS induced by LPS was at least partially produced through the Nox family. However, RRx-001 inhibited the level of ROS by reducing the expression levels of Nox1, Nox2, and Nox4 in microglia, suggesting that reducing the expression of Nox isoforms and inhibiting the production of ROS may also be another effective cause for RRx-001 to alleviate neuroinflammation.

## Conclusion

In short, the present study demonstrated that RRx-001 had an inhibitory effect on microglial activation and neuroinflammation induced by LPS through targeting TAK1. The inhibition of inflammation played a role in RRx-001s neuroprotective effects against LPS-induced neuronal toxicity. Thus, in addition as an anti-cancer drug, RRx-001 may be a candidate for the treatment of neurological diseases where neuroinflammation is implicated.

## Data Availability

The original contributions presented in the study are included in the article/Supplementary Materials, further inquiries can be directed to the corresponding author.

## Supplementary Material

Supplementary Figure S1 The drug toxicity of RRx-001 in various cells. **(A,B)** BV2 cells were pretreated with RRx-001 at proportionate doses of 0.2, 1, and 5 µM and primary microglia at doses of 0.2, 0.4, and 0.8 µM for 12 h, followed by LPS (100 ng/mL) for 12 h. Cell viability of different treatments was measured using CCK8 assay. **(C,D)** The morphology of BV2 cells and primary microglia treated as described in **(A,B)** was observed under light using an electron microscope. Scale bar, 20 µm. **(E,F)** Primary astrocytes and primary neurons were pretreated with RRx-001 at proportionate doses of 0.2, 0.4, 0.8, 1.6, and 3.2 µM for 24 h. Cell viability of different treatments was measured using CCK8 assay. **(G)** The morphology of primary astrocytes and primary neurons treated as described in **(E,F)** was observed under the light microscope. Scale bar, 20 µm. The values from three independent experiments were shown as the means ± SEM. One-way ANOVA followed by Bonferroni's post-hoc test was used for multiple comparisons between groups, * p < 0.05, ** p < 0.01, compared with no treatment group.

Supplementary Figure S2 RRx-001 did not inhibit the expression and activity of G6PD in BV2 cells. **(A,B)** BV2 cells were pretreated with RRx-001 (5 µM) for 12 h, followed by LPS (100 ng/mL) for 12 h. After the cells were harvested, the protein levels of G6PD and β-actin were detected using a western blotting assay. The relative intensity of G6PD relative to β-actin was quantified in the bar figures. **(C)** BV2 cells were pretreated with RRx-001 (5 µM) for 12 h and followed by LPS (100 ng/mL) stimulation for 6 h. After the total RNA was extracted, the mRNA levels of G6PD were determined with qRT-PCR. **(D)** The concentration of NADPH from BV2 cells treated as described in A was measured using EnzyChrom^TM^ NADP^+^/ NADPH assay kit. The values from three independent experiments were shown as the means ± SEM. One-way ANOVA followed by Bonferroni's post-hoc test was used for multiple comparisons between groups, and no significant differences were detected in any of the groups.
