# Supplementary figures and images for: RRx-001 Exerts Neuroprotection Against LPS-Induced Microglia Activation and Neuroinflammation Through Disturbing the TLR4 Pathway
Source: Front Pharmacol. 2022 Apr 6;13:889383. doi: 10.3389/fphar.2022.889383 (PMC9020799; doi:10.3389/fphar.2022.889383)

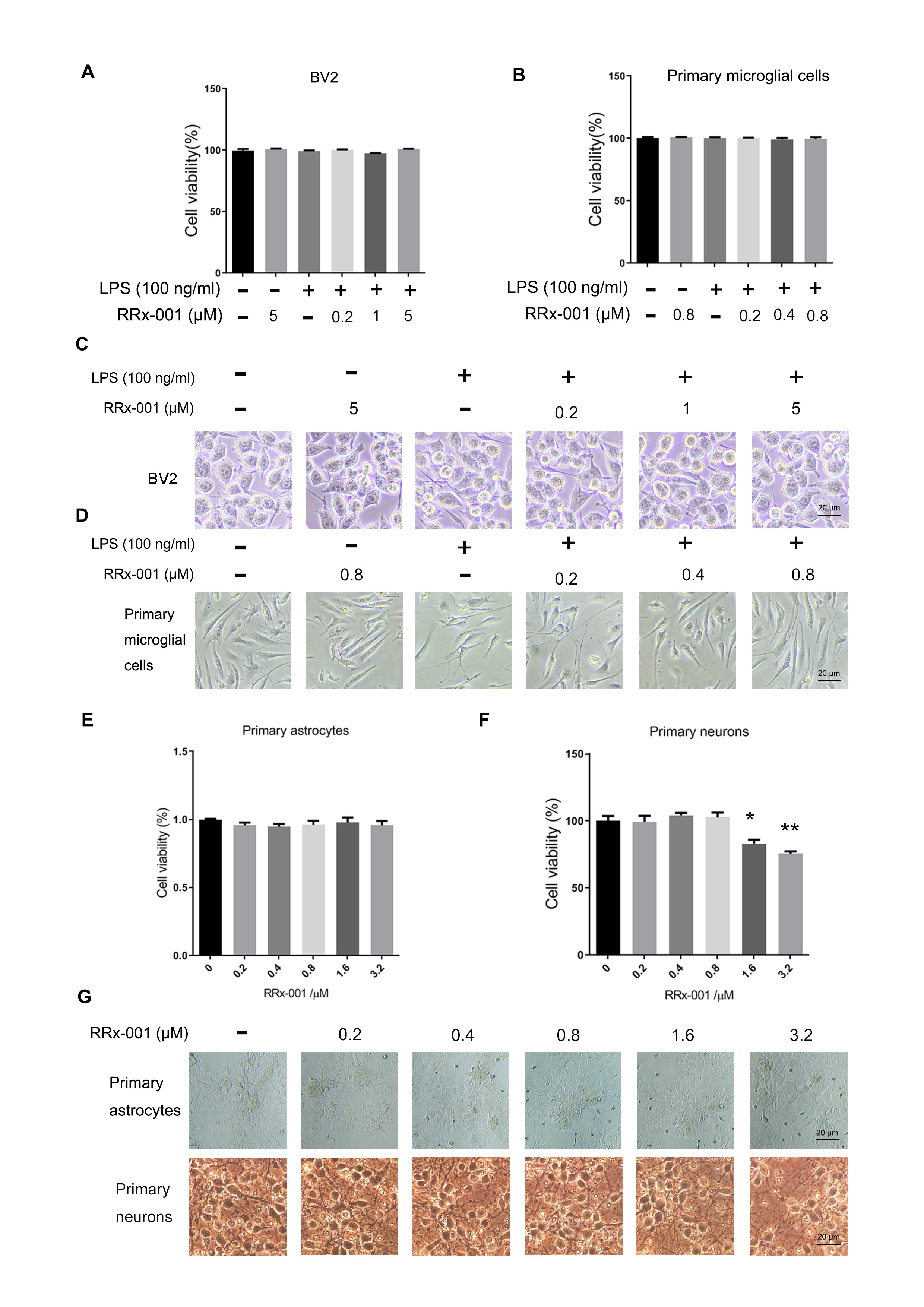

Supplement: Supplementary file 1 [file Image1.JPEG]

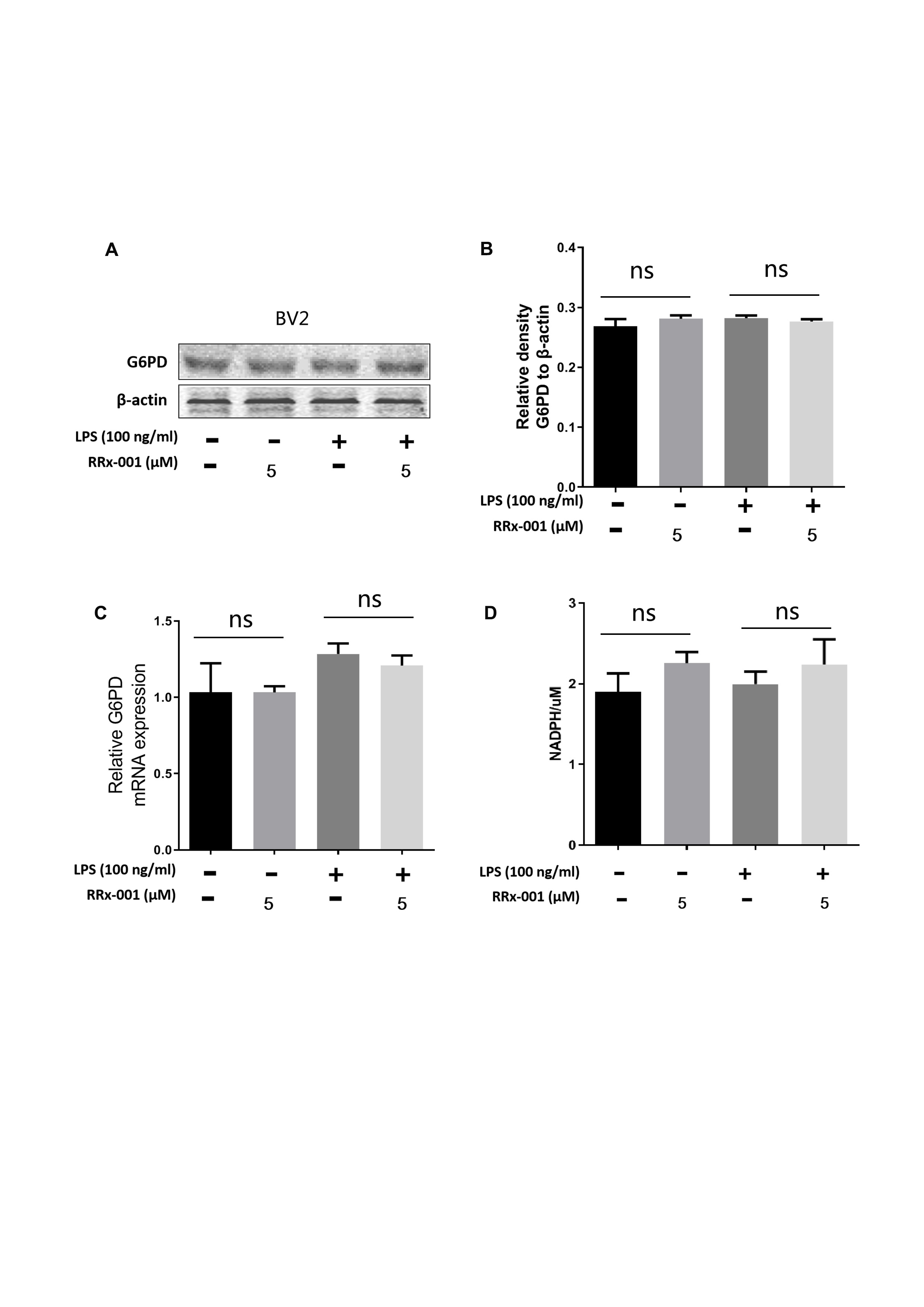

Supplement: Supplementary file 2 [file Image2.JPEG]
